# Unveiling chiral amino acid alterations and glycine dysregulation in late-life depression through targeted metabolomics

**DOI:** 10.3389/fpsyt.2025.1558796

**Published:** 2025-05-12

**Authors:** Mingxia Liu, Weigang Pan, Jing He, Sihai Ling, Yi He, Jian Yang, Peixian Mao, Zuoli Sun

**Affiliations:** ^1^ Beijing Key Laboratory of Mental Disorders, National Clinical Research Center for Mental Disorders & National Center for Mental Disorders, Beijing Anding Hospital, Capital Medical University, Beijing, China; ^2^ Laboratory for Clinical Medicine, Capital Medical University, Beijing, China

**Keywords:** late-life depression, chiral amino acid, biomarker, LC-MS/MS, targeted metabolomics

## Abstract

**Background:**

Late-life depression (LLD) is a major depressive disorder that is highly prevalent among older people, and there are currently no validated biomarkers for the diagnosis and treatment of LLD. Although dysregulated amino acid metabolism has been increasingly implicated in neuropsychiatric disorders, including LLD, most existing studies overlook the chiral nature of amino acids, potentially leading to inaccurate or incomplete findings. To address this gap, this study aimed to precisely characterize the serum chiral amino acid profiles in patients with LLD and identify potential biomarkers.

**Methods:**

Using liquid chromatography tandem mass spectrometry combined with a chiral derivatization technique, the serum levels of 34 amino acids were analyzed in 53 LLD patients and 37 healthy controls (HCs).

**Results:**

Significant alterations in both D- and L-enantiomers were observed, including reduced levels of D-methionine, D-glutamic acid, D-threonine, and L-threonine, alongside elevated glycine levels in LLD compared to HCs. The combination of D-methionine and glycine demonstrated moderate discriminatory power for distinguishing LLD from HCs, with an area under the curve of 0.71. Notably, glycine levels were significantly lower in antidepressant treatment responders than in non-responders. Additionally, D- and L-glutamic acid levels were differentially associated with specific cognitive function indicators.

**Discussion:**

These findings underscore the importance of accounting for amino acid chirality in biomarker research and highlight chiral amino acids as promising candidates for the diagnosis of LLD and the prediction of treatment response.

## 1 Introduction

Late-life depression (LLD) can be defined as major depressive disorder (MDD) occurring in individuals aged 60 years or older (1). The global prevalence of LLD is estimated at 13.3%, which is notably higher than the incidence of depression in younger populations (2). Despite the high prevalence, LLD remains a complex and multifactorial disease with no definitive biomarkers for clinical diagnosis or effective evaluation of treatment responses. Researchers have suggested that LLD may be linked to alterations in neurotransmitter systems (3), inflammatory pathways (4), neuroimaging (5), molecular genetics (6), and environmental stressors (7), but they are not yet sufficiently conclusive. Consequently, identifying objective markers for LLD has always been a significant challenge in both clinical and research settings.

Among the various pathophysiological mechanisms, amino acid neurotransmitter systems dysfunction is thought to play an important role in depression. Recent studies have reported elevated serum levels of glutamic acid (Glu), aspartic acid (Asp), and glycine (Gly) in patients with MDD, with Glu and phenylalanine (Phe) also correlating with the severity of depressive symptoms (8). In contrast, lower levels of methionine (Met), Phe, tryptophan (Trp), tyrosine (Tyr), and non-enzymatic antioxidants have been associated with drug-naive, first-episode MDD patients (9). Furthermore, branched-chain amino acids have been implicated in depression, with negative associations observed between valine (Val) and leucine (Leu) levels and depressive symptoms in young adults (10). Deficiencies in essential amino acids not only increase the risk of depression but may also impair the efficacy of pharmacological treatments. Consequently, some studies have demonstrated that amino acid supplementation, such as Trp, could potentially improve therapeutic outcomes in depressive patients (11).

Common amino acids, except Gly, generally exhibit a chiral center, which theoretically results in the presence of both L- and D-amino acid stereoisomers. It has long been assumed that the majority of amino acids in higher animals are L-enantiomers, with the D-forms being scarcely present. However, recent advancements in analytical techniques have led to the identification of various D-amino acids, such as D-serine (D-Ser), D-Asp, and D-Glu, in mammals, including humans (12). These D-amino acids have been shown to exhibit distinct biological functions and are increasingly recognized as potential clinical targets for brain disorders (13, 14). Despite the growing body of evidence, many studies have treated amino acids as a collective group, which may overlook significant variations between individual enantiomers and lead to imprecise conclusions. This is particularly relevant in the case of multifactorial and complex LLD, where only a limited number of studies have specifically investigated the role of chiral amino acids in the pathophysiology of the disorder. In 2023, our research team developed a novel, efficient liquid chromatography tandem-mass spectrometry (LC-MS/MS) method based on N^α^-(5-fluoro-2,4-dinitrophenyl)-L-leucinamide derivatization for quantifying chiral amino acids in serum. This method was initially applied to the analysis of serum chiral amino acids in LLD patients, and several potential biomarkers for LLD were identified (15).

The current study analyzed and verified the serum profiles of 17 L-amino acids, 14 D-amino acids, asparagine (Asn), Gly, and γ-aminobutyric acid (GABA) in both LLD patients and healthy controls (HCs). We assessed the differences in amino acid levels between these groups, investigated their changes before and after treatment across different response populations, and examined their correlations with symptom scores in LLD.

## 2 Materials and methods

### 2.1 Subject recruitment and sample collection

A total of 53 patients with LLD and 37 HCs were recruited from Beijing Anding Hospital, Capital Medical University, following a protocol approved by the hospital’s Human Research and Ethics Committee (2020-Scientific Research-97) (16). Written informed consent was obtained from all participants after a full explanation of the study. Inclusion criteria for LLD patients were as follows: (1) age ≥ 60 years with at least 6 years of education; (2) onset of first depressive episode after age 60; (3) diagnosis of MDD based on the Diagnostic and Statistical Manual of Mental Disorders, Fifth Edition (DSM-5); (4) a Hamilton Depression Rating Scale-17 (HAMD-17) score ≥ 17; and (5) no use of antidepressant medication at the time of enrollment. Inclusion criteria for HCs included: (1) age ≥ 60 years; (2) no history of psychiatric disorders and normal cognitive function; and (3) no use of psychotropic medications. Exclusion criteria for all participants were: (1) history of manic or hypomanic episodes; (2) comorbid dementia or other psychiatric/medical disorders; (3) severe physical illnesses (e.g., cardiovascular, hepatic, or renal disease); (4) history of brain injury; (5) substance abuse or dependence; and (6) Mini-Mental State Examination (MMSE) score ≤ 20 for those with primary education, or ≤ 24 for those with education at or above middle school level.

Patients diagnosed with LLD were treated for 8 weeks with either escitalopram or sertraline. The initial dose of escitalopram was 5–10 mg/day, with adjustments allowed up to 20 mg/day based on the patient’s clinical response and tolerability. For sertraline, the starting dose was 25–50 mg/day, with titration permitted up to 100 mg/day as needed. Depression severity was assessed using the HAMD-17, while cognitive function was evaluated using the Repeatable Battery for the Assessment of Neuropsychological Status (RBANS) at both baseline and after 8 weeks of treatment. Patients who showed at least a 50% reduction in their HAMD-17 scores post-treatment compared with the initial assessment were classified as responders, while others were categorized as non-responders (17, 18). RBANS included assessments of five cognitive domains: immediate memory, visuospatial, language, attention, and delayed memory.

For both LLD patients and HCs, the blood sampling was done during 8–12 AM after fasting for more than eight hours and participants were instructed to maintain a light diet in the days preceding sample collection. Blood samples were collected before the start of treatment (baseline) and after 8 weeks. The serum was separated by centrifugation at 1500 x g for 15 minutes and stored at -80°C until further analysis.

### 2.2 Amino acids analysis

Serum amino acid levels were measured using liquid chromatography-tandem mass spectrometry (HPLC-MS/MS) as previously described (14, 15). In brief, 50 μL of serum was mixed with 450 μL of methanol (MeOH) and vortexed for 5 minutes at 1500 rpm. The mixture was then centrifuged at 16,000 x g for 10 minutes, and the supernatant was evaporated to dryness under nitrogen. The dried residues were reconstituted in 50 μL of water and derivatized by adding 5 μL of triethylamine, followed by 50 μL of N^α^-(5-fluoro-2,4-dinitrophenyl)-L-leucinamide (1% in acetonitrile). After thorough mixing, the reaction mixture was incubated at 55 °C for 40 minutes, and the reaction was terminated by the addition of 5 μL of formic acid.

The analysis was performed on an XBridge^®^ C18 column (130 Å, 3.5 μm, 2.1 mm × 150 mm; Waters, USA) using an UltiMate 3000 HPLC system (Dionex, Sunnyvale, CA, USA) coupled with an API 4000 MS/MS system (AB Sciex, Concord, Canada). The mobile phase consisted of solvent A (0.1% formic acid in water) and solvent B (0.1% formic acid in acetonitrile), with a flow rate of 0.2 mL/min. The gradient was programmed as follows: 30% B (0–10 min), 30%-60% B (10–34 min), 60% B (34–39 min), 60%-30% B (39–40 min), followed by a 5 minutes equilibration period.

D- and L-amino acids were identified by comparing the retention times and peak area changes before and after the addition of an enantiomeric standard. Quantification was based on the peak area relative to a calibration curve derived from standards. The HPLC-MS/MS method underwent rigorous validation, as detailed in previous studies (15).

### 2.3 Statistical analysis

Data analysis was conducted using SPSS (version 26.0, SPSS Inc., Chicago, IL, USA). Descriptive statistics were used to summarize the demographic and clinical characteristics of participants. Continuous variables are presented as mean ± standard deviation (SD), and the normality of their distribution was assessed using the Shapiro-Wilk test. For group comparisons, appropriate statistical tests were selected based on distribution assumptions. For categorical variables such as gender and family psychiatric history, the chi-squared test was used. For amino acid levels that were not normally distributed, data normalization was carried out using logarithmic, quadratic, or reciprocal transformation. For continuous variables that were normally distributed or achieved normality after transformation, differences in amino acid levels between LLD patients and HCs were analyzed using Student’s t-test. For non-normally distributed continuous variables, the Mann-Whitney U test was employed to assess differences in amino acid levels between the two groups. To analyze the changes in amino acid levels from baseline to week 8 of treatment in LLD patients, the paired-samples Wilcoxon signed-rank test was used for non-normally distributed data, and the paired-samples t-test was used for normally distributed data. To evaluate the associations between amino acid concentrations and continuous variables (e.g., age and LLD symptom scores), Pearson’s correlation test was used for data following a Gaussian distribution, while Spearman’s correlation test was applied for data with a non-Gaussian distribution. To assess the relationship between amino acid levels and diagnosis, logistic regression was performed with diagnosis as the dependent variable and amino acid levels as explanatory variables, using forward stepwise selection. The receiver operating characteristic (ROC) curve and the area under the ROC curve (AUC) were used to evaluate the diagnosis value of amino acids levels in LLD patients. A repeated measures analysis of variance (RMANOVA) was employed to examine the interaction between time and group (time × group) in relation to treatment effects and amino acid levels. RMANOVA considered both time and treatment as factors. Statistical significance was set at *p* < 0.05 for all analyses.

## 3 Results

### 3.1 Demographic and clinical data of LLD patients and HCs

The demographic and clinical characteristics of 53 LLD patients and 37 HCs are summarized in **Table 1**. No significant differences were found between the LLD and HCs in terms of age (*p* = 0.076), gender (*p* = 0.524), or body mass index (BMI) (*p* = 0.791). However, significant differences were observed regarding education levels and family history of mental illness. LLD patients tended to have fewer years of education and were more likely to have a family history of mental illness compared to the HCs (*p* < 0.001). All LLD patients had moderate to severe depression, with HAMD-17 scores ranging from 17 to 34. After 8 weeks of antidepressant treatment, there was a significant reduction in the HAMD-17 scores (*p* < 0.05).

**Table 1:** Comparison of demographic and clinical variables in LLD and HCs groups.

Variables	LLD	HCs	*P*
N	53	37	–
Age (year)	66.68 ± 4.08	65.16 ± 3.74	0.076^a^
Gender (Male/Female)	18/35	15/22	0.524^b^
BMI (score)	23.95 ± 3.01	24.14 ± 2.43	0.791^a^
Education (years)	10.04 ± 3.11	13.24 ± 3.22	< 0.001^a^
Family psychiatric history	43 (81%)	37 (100%)	< 0.001^b^
Baseline HAMD-17	21.66 ± 3.64	–	–
Follow-up HAMD-17	9.24 ± 5.87	–	–

LLD, late-life depression; HCs, healthy controls; BMI, body mass index; HAMD-17, Hamilton Depression Rating Scale-17. All values are expressed as mean ± standard deviation. ^a^ Statistical *p* values derive from the t-test (t). ^b^ Statistical *p* values derive from the chi-squared test (χ^2^).

### 3.2 Comparison of amino acid levels between LLD patients and HCs

**Figure 1:**
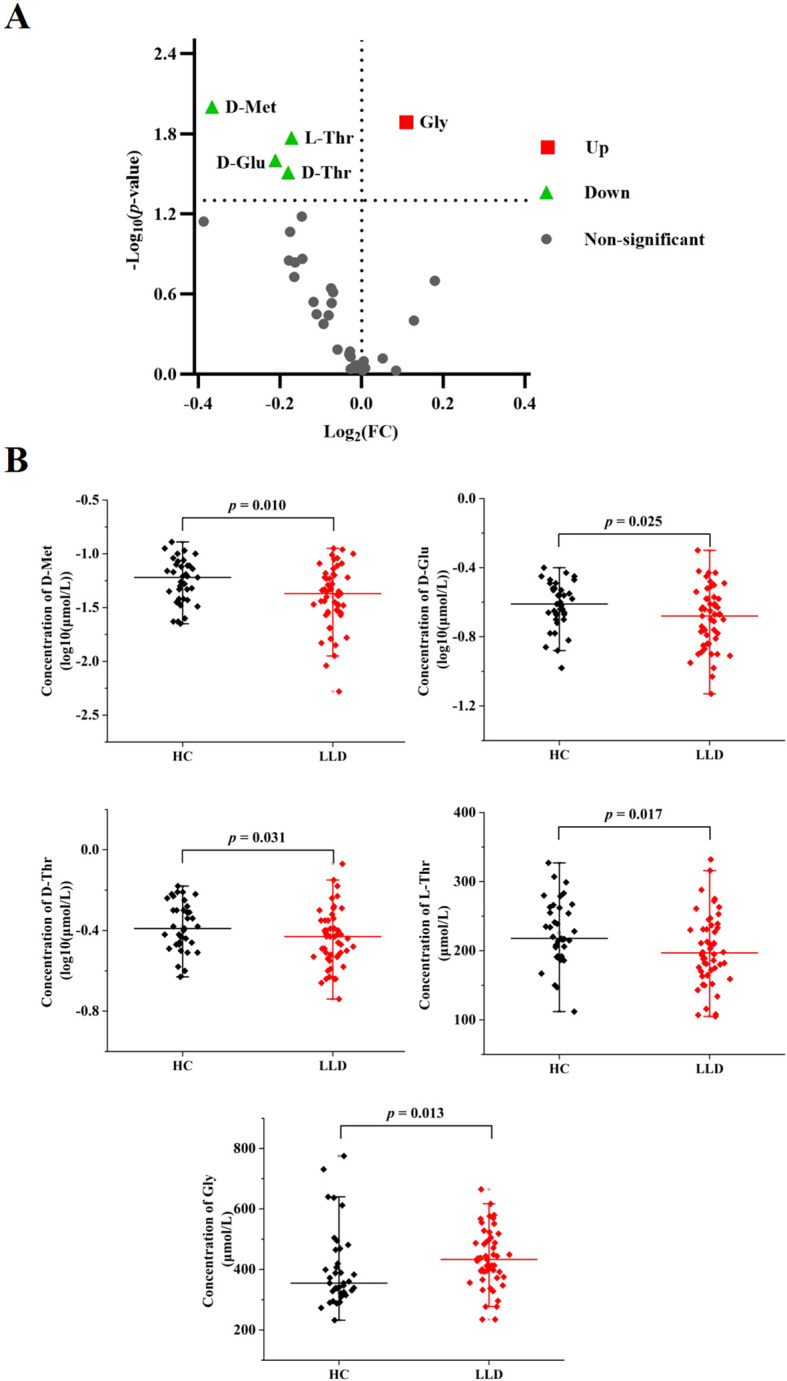
Comparison of amino acid levels between LLD patients and HCs. **(A)** Volcano plot for differential amino acids. Significantly regulated amino acids between groups determined by *p* value (*p* < 0.05). Green dots represent decreased amino acids in LDD, red dots represent increased amino acids in LDD, and gray dots represent non-significant amino acids between groups. **(B)** Serum levels of D-Met, D-Glu, D-Thr, L-Thr, and Gly in LLD patients and HCs.

There were significant differences (*p* < 0.05) between the LLD and HCs in 5/34 amino acids, that was, D-Met, D-Glu, D-threonine (D-Thr), L-Thr, and Gly (**Supplementary Table S1** and **Figure 1**). Specifically, D-Met (log10-transformed: 0.05 ± 0.03 versus 0.06 ± 0.03 μmol/L, *p* = 0.010), D-Glu (log10-transformed: 0.2228 ± 0.09 versus 0.25 ± 0.07 μmol/L, *p* = 0.025), D-Thr (log10-transformed: 0.38 ± 0.13 versus 0.43 ± 0.12 μmol/L, *p* = 0.031), and L-Thr (200.49 ± 50.15 versus 225.84 ± 46.52 μmol/L, *p* = 0.017) were significantly lower in LLD patients compared to HCs, while Gly levels (435.57 ± 94.09 versus 403.76 ± 128.85 μmol/L, *p* = 0.013) were significantly higher in LLD patients. However, when applying the Bonferroni correction with a critical *p* value of 0.00147 (0.05/34), none of the amino acids retained statistical significance.

### 3.3 Age-dependent changes in amino acid levels

**Figure 2:**
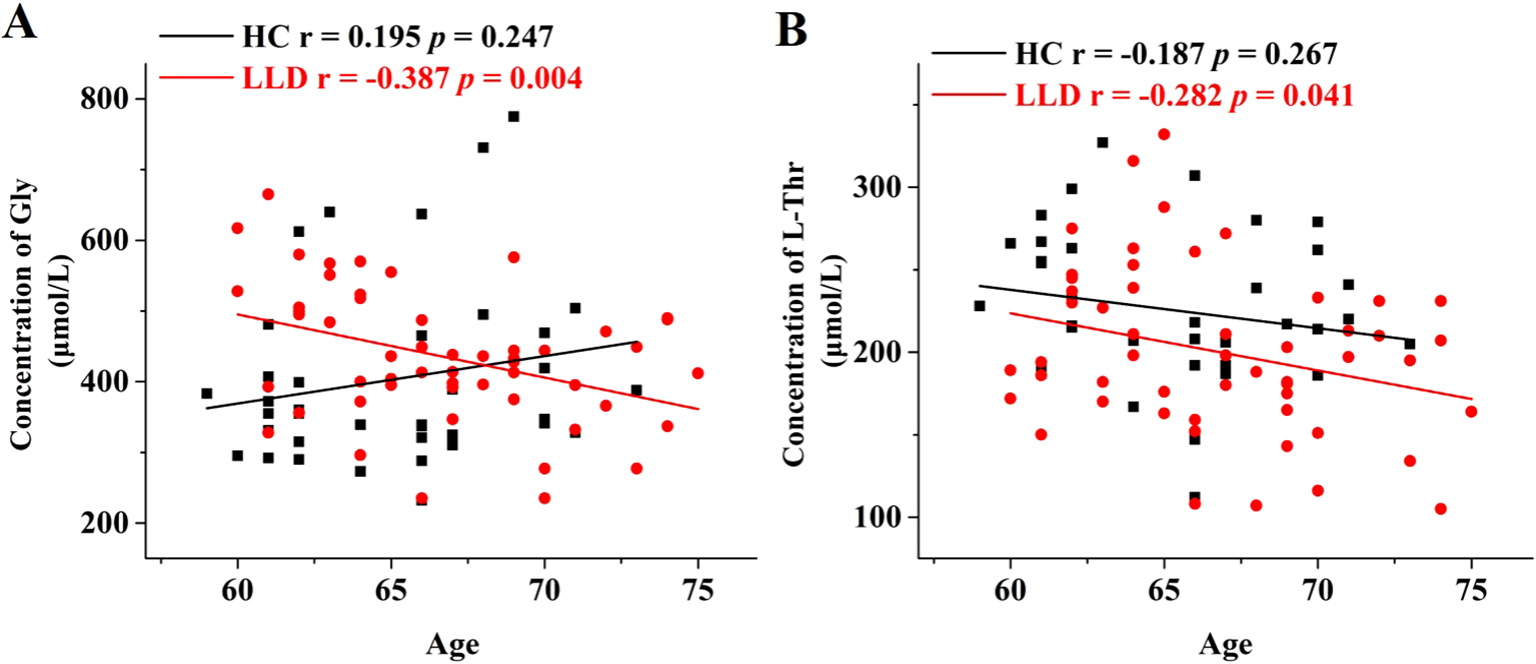
Age-dependent changes in the serum levels of Gly and L-Thr in LLD and HCs groups. Correlation analysis of age with **(A)** Gly levels and **(B)** L-Thr levels in the serum samples of HCs (black) and LLD (red).

Age-related variations of the above significantly different amino acids (D-Met, D-Glu, D-Thr, L-Thr, and Gly) were assessed using Pearson or Spearman correlation analysis (**Figure 2**). In the LLD group, significant age-related decreases were observed in the levels of Gly and L-Thr (r = -0.387, *p* = 0.004, and r = -0.282, *p* = 0.041, respectively), whereas no such correlations were found in the HCs group (r = 0.195, *p* = 0.247, and r = -0.187, *p* = 0.267, respectively). In contrast to Gly and L-Thr, the levels of D-Met, D-Glu, and D-Thr did not show significant age-related changes in either the LLD or HCs.

### 3.4 Differentiating LLD patients from HCs using amino acids

To determine the association between amino acids and diagnosis, a forward stepwise logistic regression analysis using D-Met, D-Glu, D-Thr, L-Thr, and Gly as independent variables was performed. The Nagelkerke’s R^2^ was 0.159, and the model’s coefficient was significant in the omnibus goodness-of-fit test (χ2 = 11.298, *df* = 2, *p* = 0.004). At a significance level of *p* = 0.05, Gly (*p* = 0.049) and D-Met (log10-transformed: *p* = 0.005) were retained in the model and showed a significant association with the LLD diagnosis (**Supplementary Table S2**).

**Figure 3:**
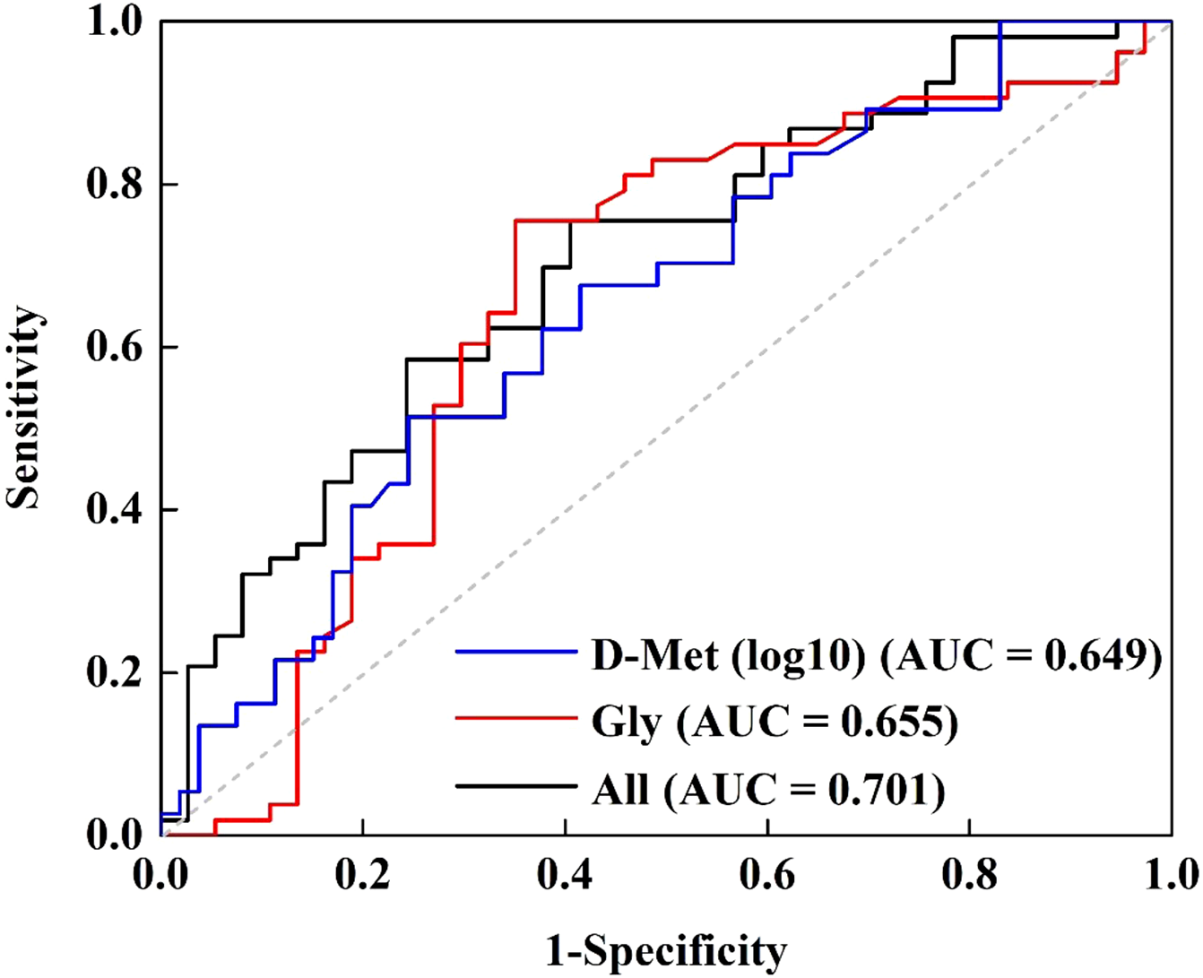
Receiver operating characteristic curve for discriminating LLD from HCs using Gly and D-Met (log10-transformed) as the diagnostic parameters.

Following these results, a receiver operating characteristic (ROC) curve analysis was performed to evaluate Gly and D-Met’s ability to distinguish LLD patients from HCs. The optimal cut-off values for Gly and D-Met (log10-transformed) were determined to be 390.500 and -1.217, with a sensitivity of 0.755 and 0.514, specificity of 0.649 and 0.755, and area under the curve (AUC) values of 0.655 and 0.649, respectively. Combining Gly and D-Met (log10-transformed) improved the AUC to 0.701 (**Supplementary Table S3**, **Figure 3**), indicating a fair discriminatory power.

### 3.5 Changes in amino acid levels with different treatment outcomes

Significant differences (*p* < 0.05) were observed in the levels of 19 out of 34 amino acids before and after antidepressant treatment (**Supplementary Table S1**). After applying the Bonferroni correction with a critical *p* value of 0.00147 (0.05/34), D-Ser, D-Thr, L-Asp, D-Asp, L-Glu, D-Glu, D-Trp, L-histidine (L-His), and D-His remained significantly altered, with all of these amino acids showing a notable increase following treatment.

To explore the relationship between amino acid levels and treatment effectiveness, LLD patients were divided into responders (n = 36 [73.47%]) and non-responders (n = 13 [26.53%]). The RMANOVA was conducted, with amino acid levels as the dependent variable and time (before and after treatment) as a factor. As shown in **Table 2**, significant time effects were observed for L-Ser, D-Ser, D-Thr, L-Asp, D-Asp, L-Glu, D-Glu, D-Trp, L-His, D-His, D-arginine (D-Arg), and L-lysine (L-Lys) (*p* < 0.05), all of which significantly increased after treatment, except for L-Lys. After applying the Bonferroni correction, only L-Asp, D-Asp, and D-His remained significant. Additionally, a significant group effect was found for Gly (*p* = 0.028), with responders exhibiting significantly lower Gly levels than non-responders. However, no statistically significant interactions between group and time were observed in this study.

**Table 2:** Results of repeated measure analysis of variance (RMANOVA) with amino acid levels as the dependent variable across time.

Variable	Responders (n=36) Mean ± SD^a^		Non-responders (n=13) Mean ± SD^a^		*P* ^b^		
	Baseline	Week 8	Baseline	Week 8	Time	Group	Interaction
L-Ser	268.93 ± 54.86	308.07 ± 78.04	286.28 ± 58.33	307.75 ± 57.69	**0.043**	0.462	0.601
D-Ser	3.21 ± 0.89	3.99 ± 1.39	2.87 ± 0.75	3.31 ± 0.69	**0.003**	0.096	0.688
L-Ala	600.72 ± 131.66	663.54 ± 186.54	696.04 ± 168.44	705.82 ± 206.59	0.376	0.121	0.451
D-Ala	2.55 ± 0.94	3.00 ± 1.22	2.64 ± 1.29	2.85 ± 1.06	0.090	0.948	0.841
L-Pro	321.79 ± 87.33	348.32 ± 138.43	354.65 ± 132.54	340.06 ± 111.49	0.831	0.752	0.572
D-Pro	0.84 ± 0.28	0.87 ± 0.37	0.80 ± 0.32	0.98 ± 0.45	0.198	0.896	0.284
L-Val	424.68 ± 104.66	448.08 ± 150.34	454.74 ± 143.33	455.46 ± 132.84	0.706	0.609	0.763
D-Val	0.39 ± 0.15	0.43 ± 0.12	0.37 ± 0.12	0.42 ± 0.12	0.081	0.685	0.932
L-Thr	196.62 ± 52.22	218.08 ± 70.26	205.45 ± 45.94	222.99 ± 56.07	0.134	0.634	0.879
D-Thr	0.39 ± 0.14	0.47 ± 0.15	0.37 ± 0.10	0.45 ± 0.15	**0.004**	0.577	0.747
L-Ile	143.13 ± 52.96	138.83 ± 69.25	154.29 ± 60.21	128.03 ± 53.84	0.144	0.957	0.462
D-Ile	0.09 ± 0.07	0.10 ± 0.05	0.08 ± 0.05	0.08 ± 0.04	0.815	0.305	0.325
L-Leu	213.37 ± 72.91	221.43 ± 96.41	244.98 ± 94.88	223.01 ± 81.83	0.674	0.466	0.366
D-Leu	0.20 ± 0.08	0.23 ± 0.05	0.21 ± 0.07	0.20 ± 0.06	0.293	0.551	0.149
L-Asp	27.21 ± 10.14	36.81 ± 14.92	31.36 ± 17.49	44.69 ± 17.53	**<0.001**	0.184	0.462
D-Asp	0.37 ± 0.12	0.46 ± 0.14	0.38 ± 0.14	0.51 ± 0.17	**<0.001**	0.596	0.456
L-Glu	96.87 ± 35.60	126.68 ± 56.20	121.18 ± 39.97	147.49 ± 59.38	**0.011**	0.052	0.683
D-Glu	0.21 ± 0.09	0.26 ± 0.09	0.24 ± 0.09	0.31 ± 0.12	**0.004**	0.176	0.893
L-Trp	59.51 ± 24.92	59.66 ± 34.57	62.52 ± 29.05	56.62 ± 33.71	0.636	0.999	0.618
D-Trp	0.05 ± 0.02	0.06 ± 0.02	0.05 ± 0.02	0.05 ± 0.02	**0.012**	0.537	0.321
L-Met	38.77 ± 15.12	41.65 ± 18.09	44.88 ± 16.15	41.23 ± 16.92	0.829	0.469	0.405
D-Met	0.05 ± 0.03	0.06 ± 0.03	0.05 ± 0.02	0.05 ± 0.02	0.105	0.53	0.121
L-His	109.43 ± 20.02	133.56 ± 33.75	119.41 ± 28.22	133.13 ± 24.42	**0.002**	0.402	0.467
D-His	0.12 ± 0.05	0.16 ± 0.04	0.13 ± 0.05	0.15 ± 0.04	**<0.001**	0.955	0.369
L-Phe	117.16 ± 41.52	120.97 ± 64.59	135.22 ± 55.99	132.55 ± 77.81	0.964	0.318	0.796
D-Phe	0.13 ± 0.05	0.15 ± 0.05	0.13 ± 0.06	0.14 ± 0.04	0.194	0.890	0.629
L-Arg	123.40 ± 34.97	131.73 ± 40.46	126.77 ± 39.80	125.56 ± 35.51	0.598	0.867	0.595
D-Arg	0.30 ± 0.12	0.33 ± 0.10	0.29 ± 0.10	0.33 ± 0.08	**0.015**	0.974	0.93
L-Tyr	49.62 ± 27.27	56.55 ± 27.41	57.37 ± 27.24	78.66 ± 27.41	0.854	0.306	0.901
L-Lys	55.00 ± 32.25	45.90 ± 31.41	61.40 ± 30.70	42.61 ± 28.92	**0.007**	0.861	0.330
L-Gln	998.97 ± 181.06	1195.99 ± 267.57	1078.13 ± 252.83	1078.27 ± 221.94	0.077	0.707	0.087
Asn	98.26 ± 22.70	110.24 ± 27.64	105.76 ± 21.84	109.94 ± 27.24	0.119	0.530	0.375
Gly	433.03 ± 102.69	436.57 ± 77.57	475.40 ± 122.12	469.02 ± 99.70	0.086	**0.028**	0.769
GABA	0.65 ± 0.40	0.77 ± 0.54	0.65 ± 0.35	0.72 ± 0.41	0.054	0.863	0.544

LLD, late-life depression; Ser, serine; Ala, alanine; Pro, proline; Val, valine; Thr, threonine; Ile, isoleucine; Leu, leucine; Asp, aspartic acid; Glu, glutamic acid; Trp, tryptophan; Met, methionine; His, histidine; Phe, phenylalanine; Arg, arginine; Tyr, tyrosine; Lys, lysine; Gln, glutamine; Asn, asparagine; Gly, glycine; GABA, γ-aminobutyric acid. ^a^ Values are expressed as mean ± standard deviation (SD). The concentration unit of all amino acids is presented as μmol/L. ^b^ Significant *p* values are in bold type.

### 3.6 Associations of amino acid levels and LLD symptom scores

**Figure 4:**
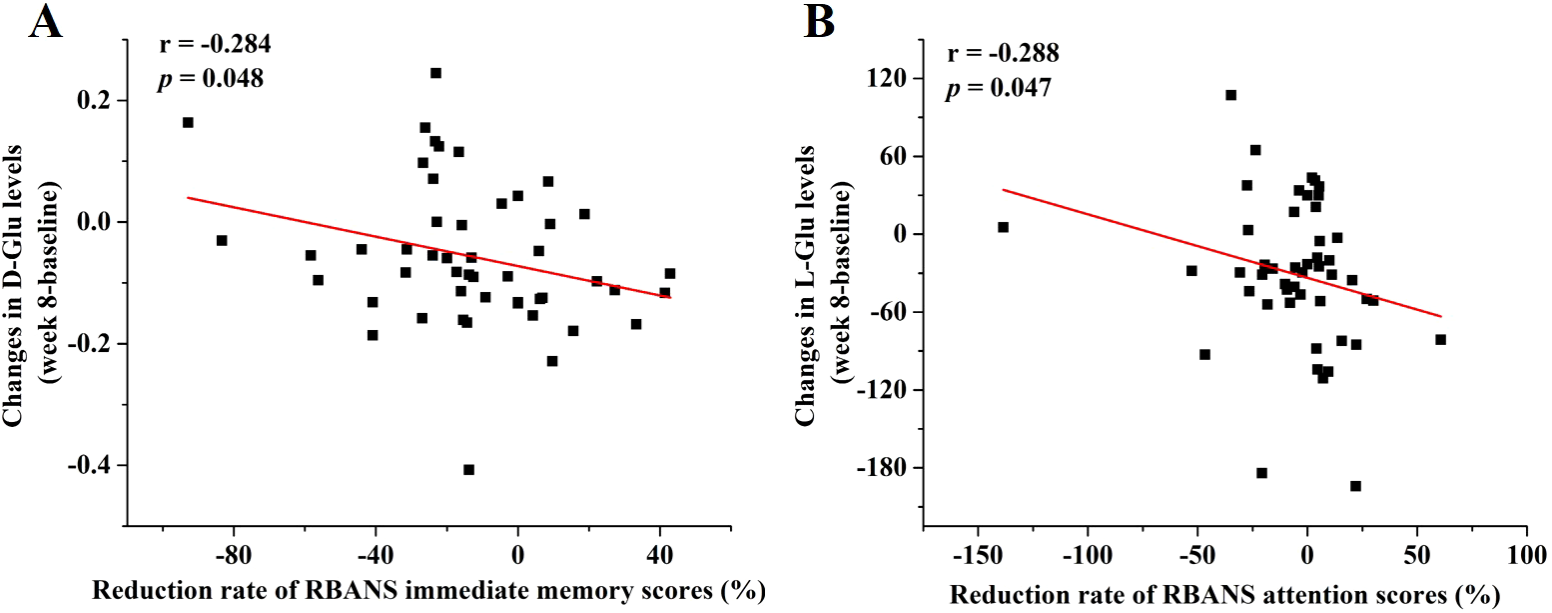
Associations between changes in amino acid levels and reduction rate of RBANS scores. The regression curves of **(A)** changes in D-Glu levels and reduction rate of RBANS immediate memory scores and **(B)** changes in L-Glu levels and reduction rate of RBANS attention scores.

To assess the relationship between changes in key amino acid levels and improvements in LLD symptoms as measured by the HAMD-17 and RBANS scales after 8 weeks of antidepressant treatment, Pearson or Spearman correlation analysis was performed. As shown in **Figure 4**, changes in D-Glu levels were significantly correlated with reductions in the RBANS immediate memory scores (r = -0.284, *p* = 0.048), and changes in L-Glu levels were significantly associated with reduced scores for RBANS attention scores (r = -0.288, *p* = 0.047). No significant correlations were found between other amino acids of interest and LLD symptom scores.

## 4 Discussion

The primary findings of this study were summarized as follows: 1) Statistical analysis identified significant differences (*p* < 0.05) in the concentrations of 5 out of 34 amino acids between LDD patients and HCs. Serum levels of D-Met, D-Glu, D-Thr, and L-Thr were notably lower, whereas Gly levels were elevated in the LDD groups compared to HCs (**Supplementary Table S1**, **Figure 1**). 2) Gly and L-Thr exhibited significant age-dependent declines in LDD patients (**Figure 2**). 3) The combination of D-Met and Gly exhibited moderate discriminatory ability to distinguish LDD patients from HCs, with an AUC of 0.701 (**Figure 3**). 4) Following antidepressant treatment, levels of L-Asp, D-Asp, and D-His significantly increased. Moreover, Gly levels were significantly lower in responders compared to non-responders (**Table 2**). 5) Significant correlations were observed between reductions in RBANS scores and the levels of D-Glu and L-Glu (**Figure 4**).

This study revealed a significant elevation in serum Gly levels among LLD patients compared to HCs. These findings aligned with prior research, which also reported increased plasma and serum Gly levels in individuals with depression (8, 19). Additionally, a notable group effect was identified for Gly, with responders displaying significantly lower Gly levels than non-responders. Supporting this, Hebbring et al. conducted a metabolomic analysis of plasma samples categorized by escitalopram response or remission status, demonstrating a negative association between Gly levels and treatment outcomes (20). Further investigation by Hebbring et al. included genotyping tag single-nucleotide polymorphisms (SNPs) related to genes involved in Gly synthesis and degradation. This analysis identified the rs10975641 SNP in the Gly dehydrogenase gene as significantly associated with treatment outcome phenotypes. Elevated Gly levels may contribute to the development of depression through their role as co-agonists of N-methyl-D-aspartate (NMDA) receptors (8). NMDA receptors, a subtype of ionotropic glutamate receptors, exhibit high calcium permeability and are primarily activated by Glu and Asp, with Gly acting as a co-agonist (21). Impaired NMDA receptor function is linked to cognitive deficits, while excessive activation can result in excitotoxicity and neuronal damage. This may correspond to our finding that the levels of Gly and its precursor, L-Thr, decrease with age in the LLD group, paralleling the decline in cognitive function. Overactivation of NMDA receptors leads to excessive calcium influx, which disrupts mitochondrial function, promotes oxidative stress through increased reactive oxygen species, and activates pro-inflammatory pathways, ultimately causing tissue and organ damage (22). Although physiological activation of the NMDA receptor is necessary for cell survival, overactivation is a signal for cell death (23). As a result, dysregulated expression and dysfunction of NMDA receptors have been associated with a range of neuropsychiatric disorders, such as autism spectrum disorders, epilepsy, schizophrenia, and related conditions (24).

Our findings indicated that D-Met levels were significantly reduced in the serum of LLD patients, with notable increases observed after antidepressant treatment. Interestingly, previous studies have detected a significant decrease in Met levels in depression patients (25, 26). Met, an essential sulfur-containing amino acid, has antioxidative activity. Wang et al. demonstrated that the anti-inflammatory mechanism of L-Met involves inhibiting nuclear factor-κB activation and upregulating glutathione S-transferase (27). In LLD patients, the reduction in Met might be linked to the abnormal pro-inflammatory state. Notably, Met is also associated with NMDA receptor regulation. Bilen et al. found that Met promotes stress resilience and alleviates social avoidance behavior by decreasing cortical NMDA receptor expression and activity through an epigenetic mechanism involving histone methylation (28). Furthermore, it is worth highlighting that L-Met is the immediate precursor of S-adenosyl-methionine (SAMe), a crucial methyl donor involved in DNA methylation and brain monoamine synthesis, both of which are key processes in mood regulation (29). SAMe has been shown to have significant antidepressant effects, and lower concentrations of SAMe have been reported in the cerebrospinal fluid (CSF) of severely depressed individuals. SAMe administration leads to increased CSF SAMe levels and improvements in depressive symptoms (30). The recent study by Sarkisova et al. revealed that L-Met treatment produced effects comparable to those of established antidepressants like imipramine and fluoxetine in WAG/Rij rats (31). However, research on the biomechanism and therapeutic potential of D-Met in depression remains underexplored. Given the diverse roles of Met isomers in various biological processes-ranging from the growth depression of broilers (32) to enzymatic activities in human erythrocytes (33)-further investigation into the specific biological functions of D-Met in the context of depression is warranted.

In addition to Gly and Met, Glu has also exhibited significant changes with depression. Notably, a substantial decrease in D-Glu levels was observed in patients with LLD, while both L- and D-1Glu levels showed significant increases following treatment. Furthermore, these changes in Glu concentrations were significantly correlated with cognitive function. These findings are consistent with a recent study by Bian et al., which reported a significant reduction in serum Glu levels in depression patients. This result was further corroborated in chronic mild unpredictable stress (CUMS) rat models (34). L-Glu has garnered increased attention due to its crucial role in the citric acid cycle (35) and its function as a neurotransmitter in the vertebrate nervous system, which controls cognitive processes in the brain (36). In contrast, the physiological role of D-Glu remains less understood. While D-Glu has been identified in bacterial cell walls (37), rat tissues (38), and human blood (14), its functions are not well-characterized. Recent research has shown that both D-Glu and L-Glu can induce transporter-mediated presynaptic autoinhibition of neurotransmitter release (39). Additionally, D-Glu has been explored as a potential modulator of hormonal secretion (40). Our study found that D-Glu and L-Glu were differentially associated with specific cognitive function indicators on the RBANS, with D-Glu correlating with immediate memory scores and L-Glu with attention scores. This novel finding underscores the potential role of chiral amino acid metabolism in cognitive dysfunction in LLD and highlights the importance of examining amino acids from the perspective of their chiral enantiomers. Although direct evidence linking RBANS scores to glutamic acid remains limited, prior studies support the involvement of glutamatergic signaling in cognitive processes. Glutamatergic and GABAergic systems contribute to memory formation and learning processes via long-term potentiation (LTP). *In vitro* studies have demonstrated that manipulating GABAergic and glutamatergic transmission through receptor-specific ligands affects LTP. For instance, blocking glutamatergic transmission reduces LTP and impairs learning, whereas blocking GABAergic transmission facilitates LTP and learning (41). Animal studies have shown that forebrain concentrations of glutamate and glutamine were found to increase after passive avoidance learning, suggesting glutamate/glutamine synthesis during the learning process (42). Furthermore, glutamate levels positively correlate with learning success, while GABA levels negatively correlate with learning performance in the anterior cingulate cortex during reward-based learning paradigms (43). Recent research also indicates that disruptions in both GABAergic and glutamatergic systems impair memory and relearning ability (41, 44). These findings are consistent with our observation that Glu levels are decreased in LLD, and the change in Glu concentration before and after treatment is negatively correlated with the reduction rate of RBANS scores. Together, these results suggest that glutamatergic signaling may play a critical role in the cognitive dysfunction associated with LLD.

Thr levels were significantly reduced in the serum of patients with LLD, with a notable increase in D-Thr observed following antidepressant treatment. Reductions in Thr levels have also been reported in the serum of patients with treatment-resistant depression (TRD) (45), as well as in the medial prefrontal cortex, plasma, and urine of animal models of depression and TRD (46–48). However, these findings are not entirely consistent, as increased Thr levels have been observed in the serum of patients with MDD and postpartum depression (49, 50). Thr is an essential amino acid crucial for the nervous system, capable of crossing the blood-brain barrier and being converted into Gly (50). Collectively, these studies suggest that Thr may play an important role in the pathophysiology and treatment of depression, warranting further investigation.

In addition to the aforementioned changes, significant differences were also found in amino acid levels before and after antidepressant treatment. Specifically, significant differences were observed in the levels of 19 out of 34 amino acids, and D-Ser, D-Thr, L-Asp, D-Asp, L-Glu, D-Glu, D-Trp, L-His, and D-His remained significantly altered after applying the Bonferroni correction. Notably, all of these amino acids showed a marked increase following treatment, which is consistent with our previous findings (51). These results suggest that antidepressant therapy-specifically treatment with Selective Serotonin Reuptake Inhibitors (SSRIs) such as escitalopram and sertraline-may have measurable regulatory effects on amino acid metabolism. Several of the significantly altered amino acids, such as Glu, Asp, and D-Ser, are well known to participate in neurotransmission and neuroplasticity, potentially linking their changes to therapeutic response (52, 53). It is worth noting that the baseline levels of these significantly increased amino acids were generally lower in LLD patients compared to HCs, and these findings imply that SSRI treatment may ameliorate the dysregulation of serum metabolites in LLD by restoring or enhancing amino acid metabolism. This highlights the potential of these amino acids as biomarkers reflecting neurochemical changes associated with clinical improvement.

Several limitations of this study should be noted. First, the sample size for each group, particularly the responder and non-responder groups, was relatively small. Second, the participants were only assessed at two time points-baseline and after 8 weeks of treatment-limiting the ability to explore the dynamics of group differences over time. Third, while several amino acid biomarkers, particularly D-amino acids, were identified, the existing reports on these biomarkers are limited, and their mechanisms remain poorly understood. Therefore, further validation through additional methodologies, such as animal studies, is required. Future research should aim to recruit larger sample sizes, incorporate more frequent follow-up visits, and evaluate the clinical usefulness of these biomarkers.

## 5 Conclusions

In conclusion, this pilot study demonstrates reduced serum levels of D-Met, D-Glu, D-Thr, and L-Thr, along with an elevated level of Gly in patients with LLD. These findings suggest that the combination of D-Met and Gly may serve as a potential diagnostic indicator for LLD, while Gly levels could be used as a biomarker for evaluating treatment efficacy in this population.

## Data Availability

The original contributions presented in the study are included in the article/**Supplementary Material**. Further inquiries can be directed to the corresponding author.
