# Supplementary material for: Unveiling chiral amino acid alterations and glycine dysregulation in late-life depression through targeted metabolomics
Source: Front Psychiatry. 2025 May 12;16:1558796. doi: 10.3389/fpsyt.2025.1558796 (PMC12104294; doi:10.3389/fpsyt.2025.1558796)
Supplement: Supplementary file 1 [file Table1.docx]

Supplementary Material

Unveiling Chiral Amino Acid Alterations and Glycine Dysregulation in Late-Life Depression through Targeted Metabolomics

Mingxia Liu^#1^, Weigang Pan^#1^, Jing He^1^, Sihang Ling^1^, Yi He^1^, Jian Yang^1^, Peixian Mao^1^, Zuoli Sun^*1,2^

^1^Beijing Key Laboratory of Mental Disorders, National Clinical Research Center for Mental Disorders & National Center for Mental Disorders, Beijing Anding Hospital, Capital Medical University, Beijing, China

^2^ Laboratory for Clinical Medicine, Capital Medical University, Beijing, China

*** Correspondence:**Zuoli Sun
zuolisun83@163.com

**Table S1.** Comparison of the concentrations of amino acids between HCs, LLD-before treatment, and LLD-after treatment groups.

| Variable | Mean ± SD^a^ | |  |  | *p*^b^  (HCs vs LLD-before treatment) | *p*^c^  (LLD-before treatment vs LLD-after treatment) |
| --- | --- | --- | --- | --- | --- | --- |
|  | HCs | LLD-before treatment | LLD-after treatment |  |  |  |
| L-Ser | 288.05 ± 55.07 | 274.40 ± 53.87 | 309.53 ± 73.56 |  | 0.244 | 0.006 |
| D-Ser | 3.21 ± 0.82 | 3.15 ± 0.84 | 3.81 ± 1.23 |  | 0.740 | **<0.001** |
| L-Ala | 620.81 ± 150.15 | 624.58 ± 141.14 | 675.52 ± 190.44 |  | 0.904 | 0.091 |
| D-Ala | 2.88 ± 0.97 | 2.60 ± 1.04 | 3.01 ± 1.18 |  | 0.137 | 0.044 |
| L-Pro | 344.08 ± 90.98 | 325.35 ± 98.18 | 344.07 ± 129.90 |  | 0.363 | 0.252 |
| D-Pro | 0.93 ± 0.35 | 0.83 ± 0.29 | 0.92 ± 0.39 |  | 0.146 | 0.191 |
| L-Val | 448.08 ± 118.95 | 438.79 ± 114.17 | 455.57 ± 145.25 |  | 0.710 | 0.408 |
| D-Val | 0.43 ± 0.11 | 0.38 ± 0.14 | 0.42 ± 0.11 |  | 0.066 | 0.092 |
| L-Thr | 225.84 ± 46.52 | 200.49 ± 50.15 | 221.53 ± 70.15 |  | **0.017** | 0.053 |
| D-Thr | 0.43 ± 0.12 | 0.38 ± 0.13 | 0.46 ± 0.14 |  | **0.031** | **<0.001** |
| L-Ile | 149.96 ± 60.89 | 148.45 ± 53.63 | 137.68 ± 64.32 |  | 0.902 | 0.254 |
| D-Ile | 0.10 ± 0.05 | 0.09 ± 0.06 | 0.09 ± 0.05 |  | 0.141 | 0.711 |
| L-Leu | 227.98 ± 85.73 | 225.21 ± 78.59 | 225.75 ± 92.25 |  | 0.874 | 0.969 |
| D-Leu | 0.22 ± 0.07 | 0.20 ± 0.07 | 0.22 ± 0.05 |  | 0.086 | 0.048 |
| L-Asp | 28.66 ± 8.61 | 28.76 ± 12.24 | 38.13 ± 15.58 |  | 0.801 | **<0.001** |
| D-Asp | 0.41 ± 0.15 | 0.37 ± 0.13 | 0.47 ± 0.16 |  | 0.288 | **<0.001** |
| L-Glu | 109.08 ± 29.89 | 103.58 ± 37.77 | 131.02 ± 56.05 |  | 0.228 | **<0.001** |
| D-Glu | 0.25 ± 0.07 | 0.22 ± 0.09 | 0.28 ± 0.09 |  | **0.025** | **<0.001** |
| L-Trp | 54.43 ± 26.39 | 61.63 ± 25.86 | 59.88 ± 33.41 |  | 0.200 | 0.726 |
| D-Trp | 0.06 ± 0.02 | 0.05 ± 0.02 | 0.06 ± 0.02 |  | 0.187 | **<0.001** |
| L-Met | 42.69 ± 16.13 | 40.97 ± 15.33 | 42.34 ± 18.58 |  | 0.655 | 0.642 |
| D-Met | 0.06 ± 0.03 | 0.05 ± 0.03 | 0.06 ± 0.03 |  | **0.010** | 0.005 |
| L-His | 114.52 ± 23.91 | 113.72 ± 23.09 | 134.19 ± 32.48 |  | 0.874 | **<0.001** |
| D-His | 0.13 ± 0.04 | 0.13 ± 0.05 | 0.16 ± 0.04 |  | 0.293 | **<0.001** |
| L-Phe | 114.54 ± 48.77 | 125.16 ± 45.77 | 127.05 ± 66.34 |  | 0.398 | 0.850 |
| D-Phe | 0.14 ± 0.04 | 0.13 ± 0.05 | 0.15 ± 0.05 |  | 0.420 | 0.063 |
| L-Arg | 126.51 ± 38.66 | 126.68 ± 37.04 | 131.53 ± 38.70 |  | 0.936 | 0.434 |
| D-Arg | 0.32 ± 0.12 | 0.30 ± 0.11 | 0.34 ± 0.10 |  | 0.355 | 0.003 |
| L-Tyr | 68.66 ± 44.47 | 52.49 ± 27.41 | 61.13 ± 65.56 |  | 0.072 | 0.953 |
| L-Lys | 54.79 ± 28.95 | 56.78 ± 31.46 | 45.10 ± 29.37 |  | 0.761 | 0.008 |
| L-Gln | 1027.03 ± 199.51 | 1019.25 ± 198.54 | 1170.52 ± 275.53 |  | 0.856 | 0.003 |
| Asn | 103.25 ± 23.27 | 101.22 ± 22.17 | 110.72 ± 28.65 |  | 0.676 | 0.037 |
| Gly | 403.76 ± 128.85 | 435.57 ± 94.09 | 472.09 ± 114.22 |  | **0.013** | 0.035 |
| GABA | 0.66 ± 0.47 | 0.65 ± 0.38 | 0.75 ± 0.50 |  | 0.916 | 0.011 |

Abbreviations: LLD, late-life depression; HCs, healthy controls; Ser, serine; Ala, alanine; Pro, proline; Val, valine; Thr, threonine; Ile, isoleucine; Leu, leucine; Asp, aspartic acid; Glu, glutamic acid; Trp, tryptophan; Met, methionine; His, histidine; Phe, phenylalanine; Arg, arginine; Tyr, tyrosine; Lys, lysine; Gln, glutamine; Asn, asparagine; Gly, glycine; GABA, γ-aminobutyric acid. ^a^ Values are expressed as mean ± standard deviation. The concentration unit of all amino acids is presented as μmol/L. ^b^ HCs vs LLD-before treatment, and significant *p* values are in bold type. ^c^ LLD-before treatment vs LLD-after treatment, and *p* values **<** 0.001 are in bold type.

**Table S2.** Results of logistic regression on diagnosis of LLD.

| **Explanatory variables** | **B** | **Exp(B)** | **Standard Error** | **Wald** | ***p*** |
| --- | --- | --- | --- | --- | --- |
| Gly | 0.005 | 1.005 | 0.002 | 3.862 | 0.049 |
| D-Met (log10) | -2.897 | 0.055 | 1.034 | 7.849 | 0.005 |
| Constant | -5.422 | 0.004 | 1.909 | 8.068 | 0.005 |

Abbreviations: LLD, late-life depression; Gly, glycine; D-Met, D-methionine.

**Table S3.** Receiver operating characteristic curve analysis of amino acids.

| **Explanatory variables** | **Cut-off** | **AUC** | **Sensitivity** | **Specificity** | **95% Confidence interval** | ***p*** |
| --- | --- | --- | --- | --- | --- | --- |
| Gly | 390.500 | 0.655 | 0.755 | 0.649 | 0.532-0.777 | 0.013 |
| D-Met  (log10) | -1.217 | 0.649 | 0.514 | 0.755 | 0.535-0.763 | 0.017 |
| All | 0.540 | 0.701 | 0.755 | 0.595 | 0.592-0.810 | 0.001 |

Abbreviations: LLD, late-life depression; Gly, glycine; D-Met, D-methionine. AUC, area under the curve.
